# High-dose radiotherapy in newly diagnosed low-grade gliomas with nonmethylated O(6)-methylguanine-DNA methyltransferase

**DOI:** 10.1186/s13014-021-01878-3

**Published:** 2021-08-19

**Authors:** Yanwei Liu, Yanong Li, Peng Wang, Li Chen, Jin Feng, Xiaoguang Qiu

**Affiliations:** 1 Department of Radiation Oncology, Beijing Tiantan Hospital, Capital Medical University, No. 119 South Fourth Ring West Road, Fengtai District, Beijing, 100070 People’s Republic of China; 2 Department of Molecular Neuropathology, Beijing Neurosurgical Institute, Capital Medical University, Beijing, 100070 People’s Republic of China; 3 National Clinical Research Center for Neurological Diseases, Beijing, 100070 People’s Republic of China

**Keywords:** Low-grade gliomas, Radiation dose, Survival, MGMT

## Abstract

**Background:**

Patients with low-grade gliomas (LGGs) harboring *O*^*6*^*-methylguanine-DNA methyltransferase* promoter nonmethylation (*MGMT*-non-pM) have a particularly short survival and are great resistance to chemotherapy. The objective of this study was to assess the efficacy of high-dose radiotherapy (RT) for LGGs with *MGMT*-non-pM.

**Methods:**

268 patients with newly diagnosed adult supratentorial LGGs from the multicenter Chinese Glioma Cooperative Group (CGCG) received postoperative RT during 2005–2018. *MGMT* promoter methylation analysis was conducted by pyrosequencing in all patients. Univariate and multivariate analysis were performed using the Cox regression to determine the prognostic factors for overall survival (OS) and progression-free survival (PFS). RT dose–response on *MGMT* status defined subtypes was analyzed.

**Results:**

On univariate analysis, the following were statistically significant favorable factors for both PFS and OS: oligodendrogliomas(*p* = 0.002 and *p* = 0.005), high-dose RT (> 54 Gy) (*p* = 0.021 and *p* = 0.029) and 1p/19q codeletion (*p* < 0.001 and *p* = 0.001). On multivariate analysis, RT dose (> 54 Gy *vs.* ≤ 54 Gy) and *IDH* mutation were independently prognostic markers for OS (HR, 0.47; 95%CI, 0.22–0.98; *p* = 0.045; and HR, 0.44; 95%CI, 0.21–0.96; *p* = 0.038, respectively) and PFS (HR, 0.48; 95%CI, 0.26–0.90; *p* = 0.022; and HR, 0.51; 95%CI, 0.26–0.98; *p* = 0.044, respectively). High-dose RT was associated with longer OS (HR, 0.56; 95%CI, 0.32–0.96; *p* = 0.036) and PFS (HR, 0.58; 95%CI, 0.35–0.96; *p* = 0.033) than low-dose RT in *MGMT*-non-pM subtype. In contrast, no significant difference in either OS (*p* = 0.240) or PFS (*p* = 0.395) was observed with high-dose RT in the *MGMT*-pM subtype.

**Conclusions:**

High-dose RT (> 54 Gy) is an independently protective factor for LGGs and is associated with improved survival in patients with *MGMT-*non-pM.

**Supplementary Information:**

The online version contains supplementary material available at 10.1186/s13014-021-01878-3.

## Background

Low-grade gliomas (LGGs) mainly refer to grade 2 by the WHO grading system and are relatively uncommon, constituting approximately 10% of all primary brain tumors in adults [1, 2]. Although often considered as “benign”, over half of these patients will develop tumor progression within 5 years and the rate of progression-free survival (PFS) at 10 years was 21–51% [3, 4]. Treatment options for LGGs include surgery, radiotherapy (RT), and/or chemotherapy. Many aspects of these treatments are controversial. A large meta-analysis, including data from phase 3 trials, confirmed that surgery followed by RT significantly improves PFS but not OS in patients with LGGs [5]. Similarly, early versus late postoperative RT improves PFS but not OS[6]. However, low-risk patients (age < 40 and total resection), not receive any treatment, have 50% risk of tumor progression 5-years postoperatively [7]. Therefore, RT is frequently utilized after surgical resection. Recently, molecular alterations, especially *isocitrate dehydrogenase 1/2* mutation (*IDH* mutation) and chromosome arm 1p/19q codeletion (1p/19q codeletion), provide important diagnostic and prognostic information that can greatly improve diagnostic accuracy and management decision-making in patients with LGGs [8]. The detections for *IDH* mutation and 1p/19q codeletion are required for LGGs classification within the revised 2016 WHO guidelines. However, *O*^*6*^*-methylguanine-DNA methyltransferase* promoter methylation (*MGMT*-pM) was rarely reported in patients with LGGs, even though it accounts for about 79–92% in these patients [9, 10]. Only one study RTOG (Radiation Therapy Oncology Group) 0424 has reported the association of *MGMT* status with the survival of patients with LGGs [11]. In this study, *MGMT* status was an independently prognostic biomarker of high-risk, LGGs treated with radiotherapy combined with concomitant and adjuvant temozolomide (TMZ) chemotherapy. A survival benefit was observed in LGGs contained a methylated *MGMT*; Similar to glioblastoma [12], *MGMT*-non-pM confers a shorter OS (3 years *vs*. not reached) and PFS (2 years *vs*. not reached) compared with *MGMT*-pM in high-risk LGGs. Unfortunately, most clinical trials tended to test new drugs (bevacizumab plus irinotecan, paclitaxel poliglumex, cilengitide combined with TMZ, temsirolimus, and procarbazine) as alternatives to TMZ for patients with *MGMT*-non-pM have failed [13–16]. However, Tini et al. reported that unmethylated-*MGMT* GBM patients benefited from a moderately escalated dose (70 Gy) of RT plus TMZ [17].

Because of the requirements for long-term follow-up for patients with LGGs, most of the studies on RT dose were conducted early, before the year 1990, and have many limitations in diagnostic (computed tomography, CT) and treatment modalities (2D planning). However, modern technology (intensity-modulated radiation therapy, IMRT and magnetic resonance imaging, MRI) can greatly improve the dose distribution of targeted field and reduce the dose of adjacent structures. Therefore, we hypothesize that RT dose escalation might be effective in LGGs with *MGMT*-non-pM based on modern technology. In this study, we analyzed retrospectively the potential benefits of high-dose RT (> 54 Gy) in 268 patients with LGGs containing the information of *MGMT* promoter methylation. Our data provide evidence for making treatment decisions and designing clinical trials.

## Materials and methods

### Patient population

268 patients with newly diagnosed adult supratentorial LGGs (WHO 2) were obtained from the multicenter Chinese Glioma Cooperative Group (CGCG) and the Chinese Glioma Genome Atlas (CGGA) in China during 2005–2018 (www.cgga.org.cn). Tumor histology was confirmed independently by two neuropathologists based on the 2007 WHO classification and the 2016 updated edition. The study protocol was approved by the Ethics Review Board of Tiantan Hospital in Beijing, China. Written informed consent was obtained from all participants. The patients had to be in the good general condition as indicated by performance score after surgery: Karnofsky Performance Scores ≥ 60. Patient characteristics (stratified by the *MGMT* status) are summarized in Table 1.

**Table 1:** Clinical features of patients with LGGs stratified by MGMT status

Characteristics	*n* (%)	MGMT-pM *n* (%)	MGMT-non-pM *n* (%)
Total	268	115 (42.9)	153 (57.1)
Sex	268 (100)		
Male	152 (56.7)	62 (40.8)	90 (59.2)
Female	116 (43.3)	53 (45.7)	63 (54.3)
Age (years)	268 (100)		
≤ 40	153 (57.2)	55 (35.9)	98 (64.1)
> 40	115 (42.8)	60 (52.2)	55 (47.8)
Histopathology	268 (100)		
A*	220 (82.1)	83 (37.7)	137 (62.3)
O	48 (17.9)	32 (66.7)	16 (33.7)
Seizure	204 (76.1)		
Yes	122 (59.5)	33 (27.0)	89 (63.0)
No	82 (40.5)	25 (30.5)	57 (69.5)
Resection	248 (92.5)		
Total	115 (46.4)	57 (49.6)	58 (50.4)
Subtotal	133 (53.6)	48 (36.1)	85 (63.9)
RT dose	268 (100)		
High	155 (57.8)	64 (41.3)	91 (58.7)
Low	113 (42.2)	51 (45.1)	62 (54.9)
Chemotherapy	260 (97.0)		
Yes	87 (33.5)	42 (48.3)	45 (51.7)
No	173 (66.5)	66 (38.2)	107 (61.8)
IDH muation	250 (93.3)		
Yes	206 (82.4)	93 (45.1)	113 (54.9)
No	44 (17.6)	8 (18.2)	36 (81.8)
1p/19q codeletion	161 (60.1)		
Yes	63 (39.1)	43 (68.3)	20 (31.7)
No	98 (70.9)	43 (43.9)	55 (56.1)

*A: including astrocytoma and oligoastrocytoma which was eliminated from the 2016 WHO classification

### Treatments

All patients underwent surgical excision and postoperative three-dimensional conformal radiotherapy (3DCRT) or IMRT. Gross tumor volume (GTV) is defined using pre-and postoperative MRI imaging (FLAIR/T2/post-contrast T1); The clinical target volume (CTV) included GTV plus a 2-cm margin. The median dose was 55.8 Gy (range, 40–66 Gy) (1.8–2.0 Gy daily, 5 days per week). The distribution of doses was shown in Additional file 1: Fig. S1. All patients received RT at 4–14 weeks (median 7.9 weeks) after surgery. The extent of resection was evaluated using preoperative and postoperative MRI. 33.5% (87/260) of patients received chemotherapy using carmustine, nimustine, or TMZ. 7 patients received radiotherapy plus concurrent chemotherapy, and 80 patients received radiotherapy plus adjuvant chemotherapy. In the first 2 years, follow-up and MRI were performed after RT every 6 months, and every 9–12 months thereafter until tumor progression.

### Pyrosequencing of *MGMT* promoter

DNA was extracted in formalin-fixed paraffin-embedded samples with a QIAamp DNA FFPE Tissue Kit (Qiagen, Hilden, Germany). Then 100 ng DNA was bisulfite converted with an Epitect Bisulfite kit (Qiagen, Hilden, Germany) according to the manufacturer’s protocol. The bisulfite-treated DNA was amplified and then sequenced by pyrosequencing. The amplification forward primer 5’-GTTTYGGATATGTTGGG ATAGTT-3’ and the biotinylated reverse primer 5’-biotin-ACRACCCAAACACTCA CCAA-3’. The methylation levels of CpG sites 75–78 were obtained with the sequencing primers 5’-GATATGTTGGGATAGT-3’ or 5’-GTTTTTAGAAYGTTTT G-3’. The methylation levels of CpG sites 76–79 were detected with a commercial *MGMT* pyrosequencing kit (Qiagen, Hilden, Germany) with a PyroMark Q24 System (Qiagen, Hilden, Germany). Standardized positive and negative controls were included in all routine pyrosequencing testing, and every test was performed by 2 experienced molecular neuropathologists together (Additional file 2: Fig. S2).

### Statistical analyses

The clinical features of the different groups were compared using the χ2 test with SPSS v22.0 (IBM, Armonk, NY, USA). OS and PFS curves were estimated by the Kaplan–Meier method and compared with the two-sided log-rank test. OS was calculated from the day of surgery to the date of the first event. The date of progression was defined as the date of the CT or MRI examination that confirmed progression or related neurologic symptoms. Cox proportional hazards regression was used to identify independently risk factors for OS and PFS. All covariates were entered and analyzed using multivariate regression. *p* < 0.05 (two-sided) was considered to indicate statistical significance.

## Results

### Patient characteristics

Among all patients enrolled in this study, the median age was 38 years (range, 14–69 years), and the male-to-female ratio was 1.31:1 (152:116). The median follow-up time was 9.12 (7.93–10.30) years. There have been 78 deaths (29.1%) and 100 recurrences (37.3%) to date. Of the 268 samples, 220 (82.1%) were astrocytoma or oligoastrocytoma (oligoastrocytoma was essentially eliminated based on the molecular pathology on the updated WHO classification in 2016) and 48 (17.9%) were oligodendrogliomas. The 5-year OS and PFS rates were 81.0% and 73.7% in all patients. The median PFS was 11.4 years, and median OS was not yet reached. The baseline characteristics of patients, stratified by MGMT status, are reported in Table 1.

### Analyses with the Cox models

A dose of 54 Gy was extensively used in clinical decisions and trials of LGGs [1, 3, 11, 18]. Depending on the dose of 54 Gy, we divided patients into 2 groups: high dose (> 54 Gy,) and low dose (≤ 54 Gy). On univariate analysis, the following were statistically significantly favorable factors for both PFS and OS: oligodendrogliomas (*p* = 0.002 and *p* = 0.005), high-dose RT (> 54 Gy) (*p* = 0.021 and *p* = 0.029) and 1p/19q codeletion (*p* < 0.001 and *p* = 0.001). Multivariate analysis of 128 valid cases showed that high-dose RT (HR, 0.47; 95% CI, 0.22–0.98; *p* = 0.045; HR, 0.48; 95% CI, 0.26–0.90; *p* = 0.022, respectively) and *IDH* mutation (HR, 0.44; 95% CI, 0.21–0.96; *p* = 0.038; HR, 0.51; 95% CI, 0.26–0.98; *p* = 0.044, respectively) were significantly prognostic factors of both OS and PFS. 1p/19q codeletion indicated a favorable prognosis despite the difference did not reach statistical significance for OS (*p* = 0.082) (Table 2).

**Table 2:** Univariate and multivariate analyses for PFS and OS based on clinical and molecular variables

Variables	n	Univariate analyses						Multivariate analyses					
		PFS			OS			PFS			OS		
		HR	95% CI	*p*	HR	95% CI	*p*	HR	95% CI	*p*	HR	95% CI	*p*
Age ≤ 40 versus > 40	268 114/154	1.09	0.73–1.64	0.681	1.05	0.66–1.65	0.847	1.05	0.56–1.97	0.877	1.40	0.70–2.80	0.346
Sex Male versus female	268 152/116	1.18	0.79–1.78	0.434	1.01	0.64–1.58	0.977	1.85	1.00–3.42	0.050	1.21	0.61–2.40	0.590
Histopathology A* versus O	268 220/48	3.17	1.54–6.55	**0.002**	3.29	1.43–7.56	**0.005**	2.16	0.77–6.09	0.144	1.92	0.59–6.19	0.276
Seizure Yes versus no	204 122/82	0.70	0.47–1.05	0.086	0.64	0.41–1.01	0.055	0.85	0.46–1.57	0.605	0.87	0.44–1.73	0.686
Resection Total versus subtotal	248 115/133	0.59	0.38–0.92	**0.018**	0.75	0.46–1.20	0.229	0.85	0.45–1.61	0.615	0.88	0.43–1.81	0.729
Chemotherapy Yes versus no	260 87/173	1.53	1.02–2.31	**0.039**	0.96	0.59–1.67	0.875	1.49	0.82–2.73	0.195	0.94	0.46–1.94	0.867
Dose > 54 Gy versus ≤ 54 Gy	268 155/113	0.63	0.42–0.93	**0.021**	0.61	0.39–0.95	**0.029**	0.48	0.26–0.90	**0.022**	0.47	0.22–0.98	**0.045**
IDH mutation Yes versus no	250 206/44	0.61	0.37–1.00	**0.049**	0.64	0.37–1.12	0.12	0.51	0.26–0.98	**0.044**	0.44	0.21–0.96	**0.038**
1p/19q co-deletion Yes versus no	161 63/98	0.31	0.17–0.59	**0.000**	0.27	0.13–0.58	**.001**	0.42	0.18–0.96	**0.039**	0.42	0.16–1.12	0.082
MGMT pM Yes versus no	268 115/153	0.83	0.54–1.30	0.421	0.67	0.39–1.14	0.135	0.78	0.43–1.43	0.421	0.64	0.31–1.33	0.243

The significance were indicated by bold font

*A including astrocytoma and oligoastrocytoma which was eliminated in the 2016 WHO classification

*PFS* progression-free survival, *OS* overall survival, *HR* hazard ratio, *CI* confidence interval

### Dose–response in patients with *MGMT*-non-pM

**Fig. 1:**
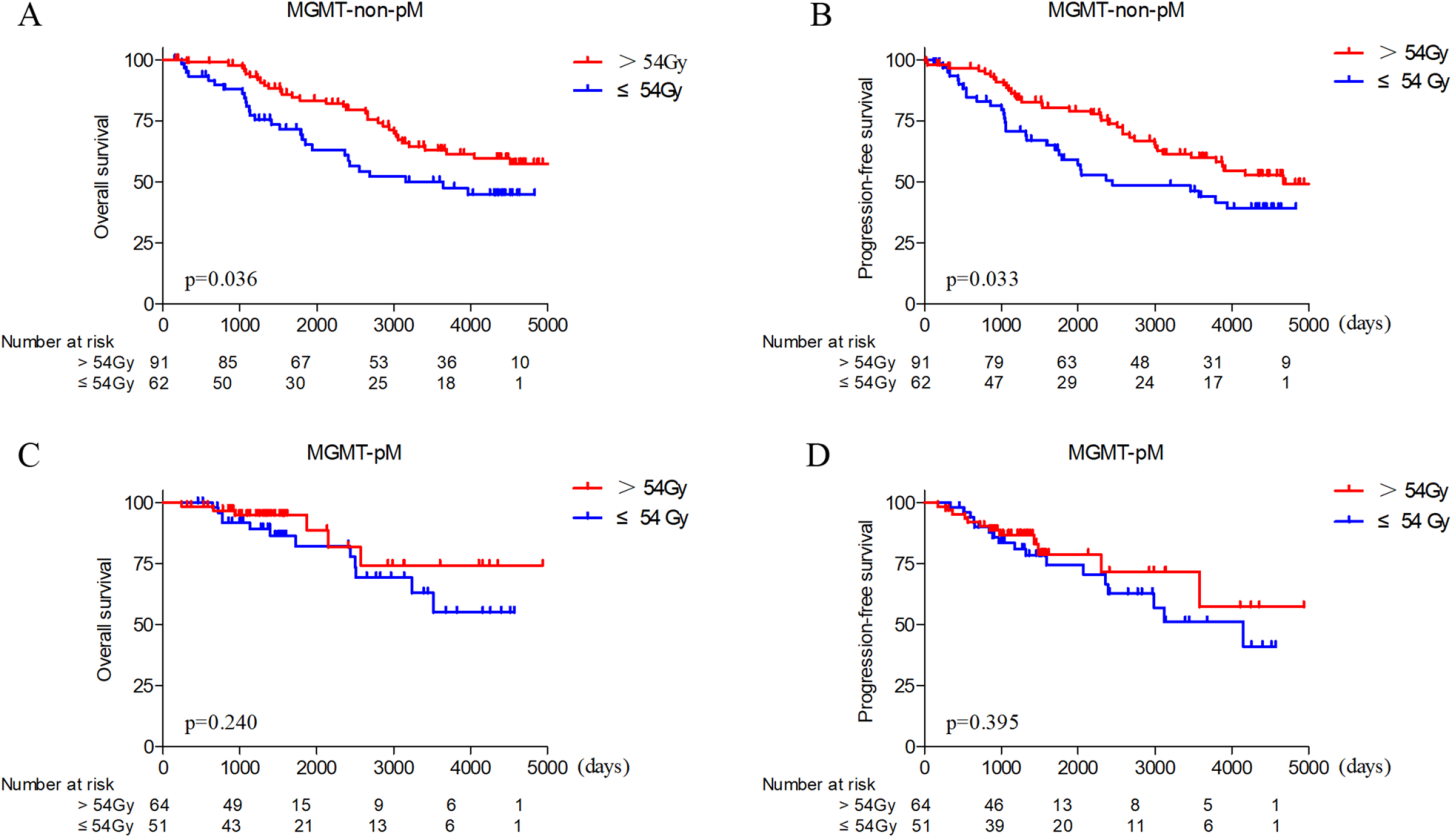
RT dose effects on *MGMT* status defined subtypes. Patients with *MGMT*-non-pM (**A**, **B**) could benefit from high-dose radiotherapy (> 54 Gy); Patients with *MGMT*-pM did not benefit from high-dose RT (**C**, **D**)

*MGMT* promoter methylation was profiled in all patients. A significant protective effect on OS and PFS with a RT dose > 54 Gy was observed in patients with *MGMT*-non-pM (HR, 0.56; 95%CI, 0.32–0.96; *p* = 0.036;; and HR, 0.58; 95%CI, 0.35–0.96;* p* = 0.033, respectively) (Fig. 1A, B), but this was not the case in patients with *MGMT*-pM (*p* = 0.240 in OS and *p* = 0.395 in PFS) (Fig. 1C, D). Most of the clinical characteristics were comparable between groups (Additional file 3: Table S1). Among 260 patients, 87 received chemotherapy (carmustine, nimustine, or TMZ). But patients with *MGMT*-pM did not receive benefit from the addition of chemotherapy (*p* = 0.195 in OS and *p* = 0.058 in PFS) (Additional file 4: Fig. S3A, B). Chemotherapy also not improved the OS (*p* = 0.697) and PFS (*p* = 0.140) in patients with *MGMT*-non-pM (Additional file 4: Fig. S3C, D).

## Discussion

Gliomas with *MGMT*-non-pM are striking resistant to chemotherapy or targeted therapy. In our study, high-dose RT (> 54 Gy) was an independently protective factor of patients with LGGs. More importantly, patients with *MGMT*-non-pM can benefit from high-dose RT, but no benefit was observed with high-dose RT in patients with *MGMT*-pM. The results showed that replacement of TMZ chemotherapy by high-dose RT might be feasible for these patients with *MGMT*-non-pM. To the best of our knowledge, this is the first report on the relationship between RT dose and *MGMT* status. *MGMT* status could serve as the primary predictor of response to RT in LGGs.

MGMT is a DNA repair protein and a marker of resistance to the first line chemotherapeutic drug (TMZ). Methylated *MGMT* resulted in reduced protein and is a strong prognostic and predictive biomarker for benefit from TMZ chemotherapy in patients with GBM, especially in elderly patients [19, 20]. Even in patients with treatment by only radiotherapy, *MGMT*-pM also confers a survival advantage [12, 21]. However, patients with *MGMT*-non-pM derive less benefit from TMZ or other alkylating agents and have shorter survival compared to those whose tumors are methylated. Though many trials have tried to test new drugs as alternatives to TMZ, none of these was effective against unmethylated GBM. However, Tini et al. reported that unmethylated-MGMT GBM patients benefited from a moderately escalated dose (70 Gy) of RT plus TMZ [17]. LGGs have relatively higher rates (75–92.5%) of *MGMT*-pM than GBM, but the association of *MGMT* status with the survival of LGGs is rarely reported. In RTOG 0424, *MGMT*-pM was found in 76% (57/75) of high-risk LGGs and was an independently prognostic biomarker based on RT and concurrent and adjuvant TMZ chemotherapy. *MGMT*-non-pM was significantly associated with worse OS and PFS than *MGMT*-pM in high-risk LGGs [11]. However, the implication of *MGMT* status concerning radio-chemotherapy sensitivity in patients with LGGs is not further studied.

Learning from the studies in GBM with *MGMT*-non-pM, we hypothesize that RT dose escalation might be effective in these refractory tumors. Earlier retrospective studies have observed a dose–response relationship in LGGs. Although these studies were retrospective and had limited sample sizes (< 150 patients), they found that high-dose RT (> 52 Gy, > 53 Gy, or even > 55 Gy) confers a survival advantage compared with those who received low-dose RT (< 52 Gy, < 53 Gy, or even < 55 Gy) [22–24]. However, two randomized trials (the European Organisation for Research and Treatment of Cancer 22,844 and the North Central Cancer Treatment Group 86-72-51) did not show an OS or PFS benefit to high-dose RT (59.4 Gy and 64.8 Gy) over low-dose RT (45 Gy and 50.4 Gy) [25, 26]. The point to emphasize here is that these studies were activated in 1985 and 1986, respectively, and patients were treated in an era with older surgical, diagnostic instrument (CT scan), and radiation techniques (2D planning). Currently, highly conformal fractionated RT techniques (IMRT or VMAT) and MRI are routinely used in clinical practice that has been a significant improvement in dose distribution of targeted field and dose limitation of adjacent structures [27]. According to National Comprehensive Cancer Network (NCCN) guideline, patients with LGGs should receive 45–54 Gy in 1.8–2.0 Gy fractions [18]. But molecular pathology provides additional diagnostic and prognostic information that can greatly improve diagnostic accuracy and management decision-making. It is suitable that consider RT dose escalation to 59.4–60 Gy for IDH wild-type LGGs. Therefore, it is needed to be reconsidered based on modern technology whether high-dose RT can obtain improved survival in some molecular subtypes. In our study, 268 patients with newly diagnosed LGGs received postoperative 3DCRT or IMRT. The RT dose is an independently prognostic factor for both OS and PFS, indicating that the survival might be further improved by increasing RT dose using modern technology. Based on histological features, high-dose RT was associated with longer OS and PFS than low-dose RT in patients with astrocytomas. In contrast, no significant difference in either OS or PFS was observed with high-dose RT in the patients with oligodendroglioma (Additional file 5: Fig. S4). Based on MGMT status, high-dose RT was associated with longer PFS and OS in the *MGMT*-non-pM subtype. In contrast, no significant difference in survival was observed with high-dose RT in the *MGMT*-pM subtype. The results showed that high-dose RT as alternatives to TMZ might be effective in LGGs with *MGMT*-non-pM. But it should be emphasized that no information on the quality of life was available in this retrospective study. Published data showed high-dose radiotherapy tended to report lower levels of functioning and more symptom burden [28]. Patients with LGGs who received RT showed a progressive decline in attentional functions compared with those who did not receive RT [29]. However, the final report from the NCCTG 86-72-51 trial showed that long-term cognitive function did not differ significantly between patients who received 50.4 Gy and those who received 64.8 Gy [25]. The impact of radiation dose on long-term quality of life, as well as neurocognitive functioning, remains to be investigated. Nevertheless, the associations of *MGMT* status with RT dose were first reported in the present study, our data is helpful in the choice of therapeutic strategy for these refractory molecular subtypes. Although confirmation by prospective trials is needed, this study is also helpful in designing clinical trials for LGGs based on *MGMT* status.

## Conclusion

High-dose RT (> 54 Gy) was an independently protective factor for patients with LGGs. Patients with *MGMT*-non-pM may have improved survival upon administration of high-dose RT. Our findings will help to define the standard of care and assist the design of prospective clinical trials for LGGs. However, the limitations of our retrospective study should be acknowledged.

## Supplementary Information

**Additional file 1: Fig. S1.** The distribution of RT doses in LGG patients

**Additional file 2: Fig. S2.***MGMT* promoter methylation was analyzed by pyrosequencing. > 10% in average was considered to be methylation

**Additional file 3.** Variables stratified by MGMT status and radiotherapy dose.

**Additional file 4: Fig. S3.** Chemotherapy effects on *MGMT* status defined subtypes. Patients with *MGMT*-pM (**A**, **B**) or *MGMT*-non-pM did not benefit from additional chemotherapy (**C**, **D**)

**Additional file 5: Fig. S4.** Radiation dose effects on histological subtypes. Patients with astrocytomas (astrocytoma and mixed oligoastrocytoma) (**A**, **B**) benefit from high-dose radiation, but not in oligodendroglioma (**C**, **D**)

## Data Availability

All data were presented in the manuscript and supplementary materials.
