# Supplementary figures and images for: High-dose radiotherapy in newly diagnosed low-grade gliomas with nonmethylated O(6)-methylguanine-DNA methyltransferase
Source: Radiat Oncol. 2021 Aug 19;16:157. doi: 10.1186/s13014-021-01878-3 (PMC8375106; doi:10.1186/s13014-021-01878-3)

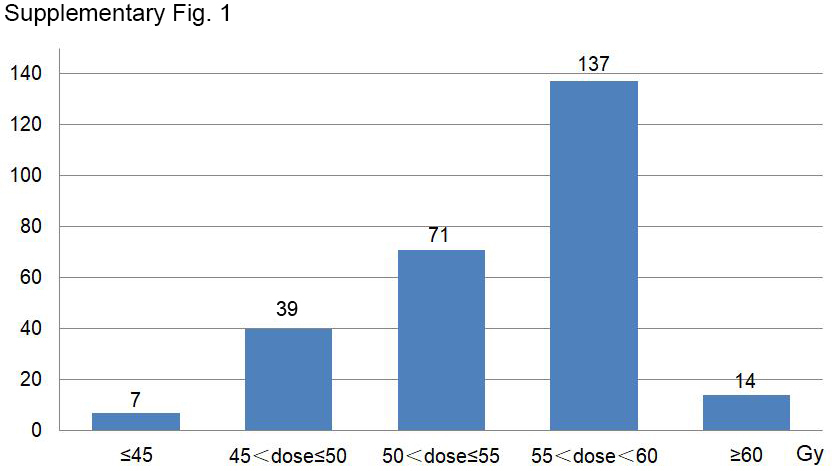

Supplement: Supplementary file 1 — Additional file 1: Fig. S1. The distribution of RT doses in LGG patients [file 13014_2021_1878_MOESM1_ESM.jpg]

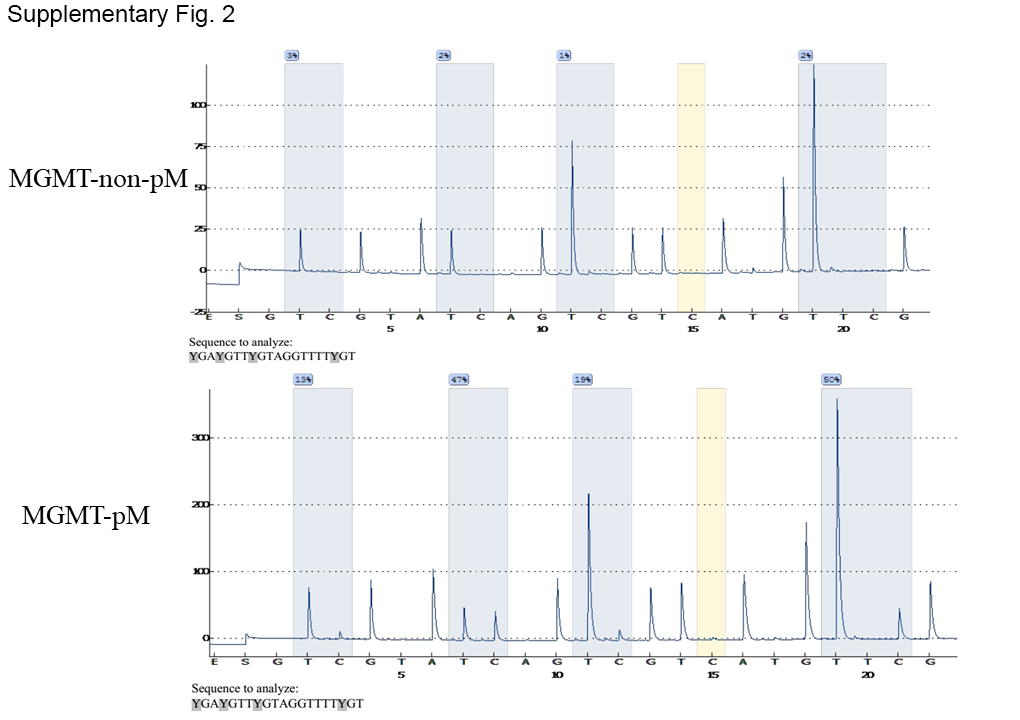

Supplement: Supplementary file 2 — Additional file 2: Fig. S2.MGMT promoter methylation was analyzed by pyrosequencing. > 10% in average was considered to be methylation [file 13014_2021_1878_MOESM2_ESM.tif]

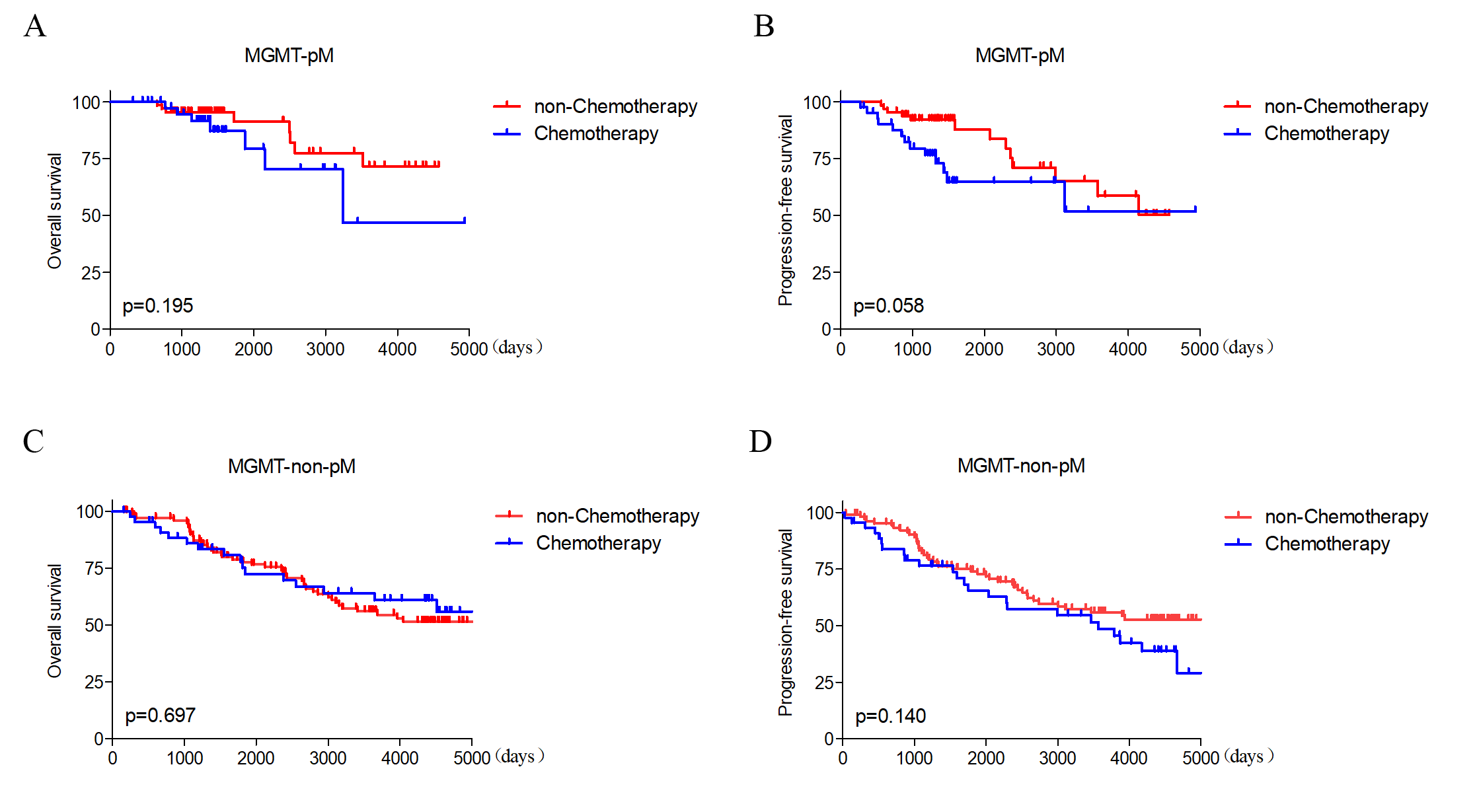

Supplement: Supplementary file 4 — Additional file 4: Fig. S3. Chemotherapy effects on MGMT status defined subtypes. Patients with MGMT-pM (A, B) or MGMT-non-pM did not benefit from additional chemotherapy (C, D) [file 13014_2021_1878_MOESM4_ESM.tif]

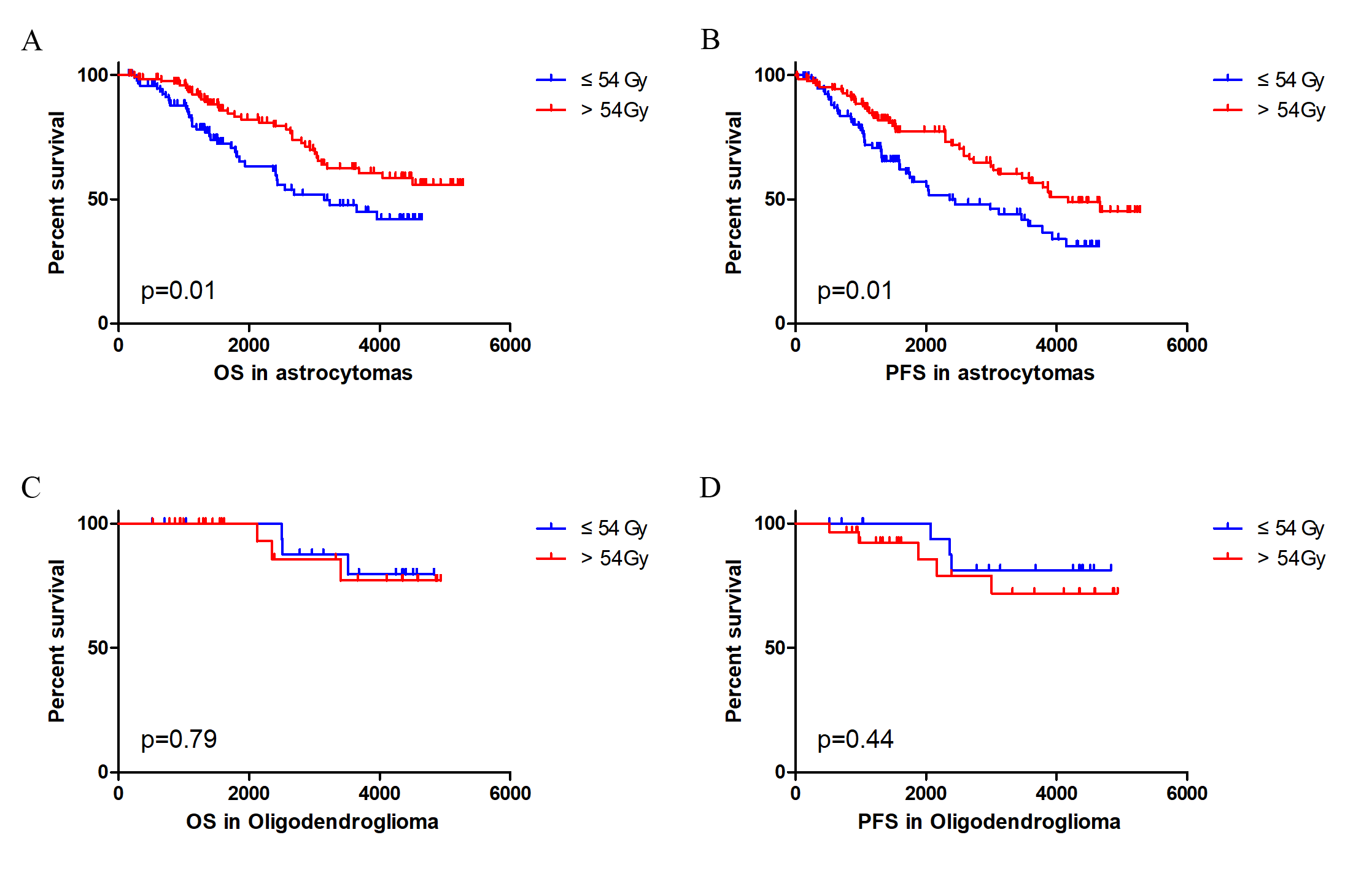

Supplement: Supplementary file 5 — Additional file 5: Fig. S4. Radiation dose effects on histological subtypes. Patients with astrocytomas (astrocytoma and mixed oligoastrocytoma) (A, B) benefit from high-dose radiation, but not in oligodendroglioma (C, D) [file 13014_2021_1878_MOESM5_ESM.tif]
