# Supplementary material for: High-dose radiotherapy in newly diagnosed low-grade gliomas with nonmethylated O(6)-methylguanine-DNA methyltransferase
Source: Radiat Oncol. 2021 Aug 19;16:157. doi: 10.1186/s13014-021-01878-3 (PMC8375106; doi:10.1186/s13014-021-01878-3)
Supplement: Supplementary file 3 — Additional file 3. Variables stratified by MGMT status and radiotherapy dose. [file 13014_2021_1878_MOESM3_ESM.docx]

**Supplementary Table 1. Variables stratified by MGMT status and radiotherapy dose**

| **Variables** | MGMT pM | | | | MGMT non-pM | | | |
| --- | --- | --- | --- | --- | --- | --- | --- | --- |
|  | n | ≤54 Gy | >54Gy | *p* | n | ≤54 Gy | >54Gy | *p* |
| **Age** | 115 | | | 0.260 | 153 | | | 0.393 |
| ≤ 40 | 55 | 21 | 34 |  | 98 | 37 | 61 |  |
| ＞40 | 60 | 30 | 30 |  | 55 | 25 | 30 |  |
| **Sex** | 115 | | | 0.355 | 153 | | | 0.315 |
| Male | 62 | 30 | 32 |  | 90 | 33 | 57 |  |
| Female | 53 | 21 | 32 |  | 63 | 29 | 34 |  |
| **Histopathology** | 115 | | | 0.835 | 153 | | | 0.592 |
| A* | 83 | 36 | 47 |  | 137 | 57 | 80 |  |
| O | 32 | 15 | 17 |  | 16 | 5 | 11 |  |
| **Seizure** | 58 | | | 0.597 | 146 | | | **0.000** |
| Yes | 33 | 20 | 13 |  | 89 | 23 | 66 |  |
| No | 25 | 13 | 12 |  | 57 | 34 | 23 |  |
| **Resection** | 105 | | | 0.699 | 143 | | | 0.301 |
| Total | 57 | 25 | 32 |  | 58 | 21 | 37 |  |
| Subtotal | 48 | 23 | 25 |  | 85 | 39 | 46 |  |
| **Chemotherapy** | 108 | | | 0.553 | 152 | | | **0.048** |
| Yes | 42 | 20 | 22 |  | 45 | 24 | 21 |  |
| No | 66 | 27 | 39 |  | 107 | 38 | 69 |  |
| **IDH mutation** | 101 | | | 1.000 | 149 | | | 1.000 |
| Yes | 93 | 41 | 52 |  | 113 | 46 | 67 |  |
| No | 8 | 4 | 4 |  | 36 | 14 | 22 |  |
| **1p/19q co-deletion** | 86 | | | 0.828 | 75 | | | 1.000 |
| Yes | 43 | 20 | 23 |  | 20 | 10 | 10 |  |
| No | 43 | 18 | 25 |  | 55 | 26 | 29 |  |
